# Human Papillomavirus 58 E7 T20I/G63S Variant Isolated from an East Asian Population Possesses High Oncogenicity

**DOI:** 10.1128/JVI.00090-20

**Published:** 2020-03-31

**Authors:** Siaw Shi Boon, Chichao Xia, Jin Yan Lim, Zigui Chen, Priscilla T. Y. Law, Apple C. M. Yeung, Miranda Thomas, Lawrence Banks, Paul K. S. Chan

**Affiliations:** a Department of Microbiology, The Chinese University of Hong Kong, Prince of Wales Hospital, Hong Kong SAR; b International Centre for Genetic Engineering and Biotechnology, Trieste, Italy; University of California, Irvine

**Keywords:** HPV58E7, V1, T20I/G63S, oncogenicity

## Abstract

Human papillomavirus (HPV) type 58 is the third most commonly detected HPV type in cervical cancer among Eastern Asians. Our previous international epidemiological studies revealed that HPV58 carrying an E7 natural variant, T20I/G63S (designated V1), was associated with a higher risk of cervical cancer. We recently showed that V1 possesses a greater ability to immortalize and transform primary cells, as well as degrading pRB more effectively, than the prototype and other common variants. In this study, we performed a series of phenotypic and molecular assays using physiologically relevant *in vitro* and *in vivo* models to compare the oncogenicity of V1 with that of the prototype and other common natural variants. Through activation of the AKT and K-Ras/extracellular signal-regulated kinase (ERK) signaling pathways, V1 consistently showed greater oncogenicity than the prototype and other variants, as demonstrated by increased cell proliferation, migration, and invasion, as well as induction of larger tumors in athymic nude mice. This study complements our previous epidemiological and molecular observations pinpointing the higher oncogenicity of V1 than that of the prototype and all other common variants. Since V1 is more commonly found in eastern Asia, our report provides insight into the design of HPV screening assays and selection of components for HPV vaccines in this region.

**IMPORTANCE** Epidemiological studies have revealed that a wild-type variant of HPV58 carrying an E7 variation, T20I/G63S (V1), is associated with a higher risk of cervical cancer. We previously reported that this increased oncogenicity could be the result of the virus’s greater ability to degrade pRB, thereby leading to an increased ability to grow in an anchorage-independent manner. In addition to this, this report further showed that this HPV variant induced activation of the AKT and K-Ras/ERK signaling pathways, thereby explaining its genuine oncogenicity in promoting cell proliferation, migration, invasion, and formation of tumors, all to a greater extent than the prototype HPV58 and other common variants.

## Abstract (precis)

Epidemiological studies have revealed that a wild-type variant of HPV58 carrying an E7 variation, T20I/G63S (V1), is associated with a higher risk of cervical cancer. We previously reported that this increased oncogenicity could be the result of the virus’s greater ability to degrade pRB, thereby leading to an increased ability to grow in an anchorage-independent manner. In addition to this, this report further showed that this HPV variant induced activation of the AKT and K-Ras/ERK signaling pathways, thereby explaining its genuine oncogenicity in promoting cell proliferation, migration, invasion, and formation of tumors, all to a greater extent than the prototype HPV58 and other common variants.

## INTRODUCTION

Among the cancer-causing or so-called high-risk human papillomavirus types (hrHPV), HPV16 and HPV18 remain the most prevalent globally. However, the distribution of other hrHPV types varies geographically. While HPV58 ranks as the fifth most common among the hrHPV types detected in cervical cancer worldwide (1), it is among the top three most prevalent hrHPV types found in East Asia (2–4) and South America (5, 6). For instance, the prevalence of HPV58 in cervical squamous cell carcinoma (SCC) was 8 to 30% in East Asia (7–10) but only 0.8 to 2.8% in other parts of the world (2). Our previous meta-analysis based on “attribution,” to minimize overcounting due to bystander coinfections, revealed that in East Asia the attribution of cervical cancer to HPV58 was 3.7-fold higher than that in other parts of the world (11).

HPV infection does not necessarily lead to carcinoma. It is a multistep process that requires persistent infection of HPV and the presence of cofactors (12–16). In general, when HPV infects primitive basal keratinocytes, the viral genes E6 and E7, which encode the respective viral oncoproteins, are expressed. The combinatorial effects of E6 and E7 allow HPV to drive cancer progression. E7 alone might be sufficient to immortalize primary cells (17) and induce a G_1_/S-like phase in the cell. These activities of E7 are complemented by those of E6, which acts to perturb the normal functions of tumor suppressors and cell polarity proteins, including p53 (18) and PSD95/Dlg/zonula-occludens-1 (PDZ) proteins (19), respectively. All of these events allow full transformation of cells and continues the cancer phenotype (20, 21). Similar to the classical hrHPVs, HPV58 E7 (HPV58E7) is able to degrade pRB (22, 23) and abrogate the G_1_/S cell cycle checkpoint, while HPV58 E6 (HPV58E6) degrades p53 (24) and PDZ proteins (25). Additionally, a recent report showed that the levels of E6 and E7 increase in correspondence with lesion progression. This leads to increased activation of the DNA damage response and to cellular genome instability (26), enhanced telomerase activity (27) and activation of oncogenic signaling pathways, including AKT (28, 29) and extracellular signal-regulated kinase (ERK) (30).

Previous epidemiological studies from our group and others have identified three HPV58E7 variants commonly found in East Asia: T20I/G63S (V1), G41R/G63D (V2), and T74A/D76E (V3) (31, 32). Of these variants, HPV58E7 V1 (58E7V1) was reported to confer a higher risk for high-grade cervical dysplasia or cervical intraepithelial neoplasia III (CIN III) and invasive cervical cancer (31, 33), while V2 and V3 carry a lower risk. Of note, a study from the eastern part of China, Zhejiang, revealed that V2, not V1, was the predominant variant found in a set of CIN II and SCC cancers (34). The discrepancy among epidemiological observations underscores the need for risk assessment by cellular and animal models. We recently showed that HPV58E7 V1 possesses a greater ability to immortalize primary epithelial cells, to induce anchorage-independent growth, and to degrade pRB than the HPV58E7 prototype (58E7P) or the V2 (58E7V2) and V3 (58E7V3) variants (22).

In this study, we extended our analyses using *in vitro* and *in vivo* models to compare the oncogenicities of the HPV58E7 prototype and the V1, V2, and V3 variants. We have established murine-derived epithelial cells constitutively expressing either the HPV58E7 prototype or the V1, V2, or V3 variant. We compared their activities in cell proliferation, invasion, and tumor formation in mice and explored the mechanisms involved.

## RESULTS

### Murine-derived keratinocytes constitutively expressed HPV58E7 prototype and its variants.

**FIG 1:**
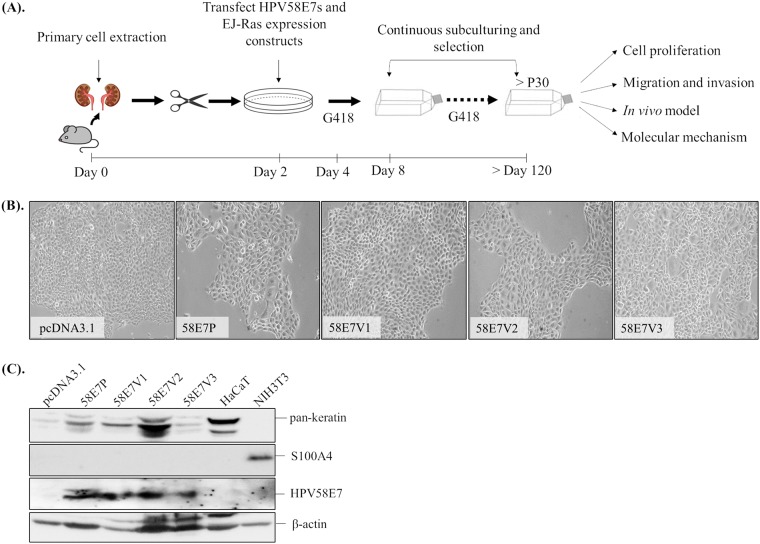
Establishment of murine-derived primary epithelial cells with constitutive expression of HPV58E7. (A) Schematic diagram showing the workflow of establishment of BRK-HPV58E7 cells. Kidneys of 9-day-old Wistar Hannover rats were extracted, excised, trypsinized, and plated onto tissue culture dishes. After overnight culture, the attached monolayer cells were transfected with plasmids expressing EJ-Ras and HPV58E7 (prototype or V1, V2, or V3 variant). The cells were cultured under Geneticin (G418) selection. Upon reaching 90% confluence, G418-resistant cells were transferred and cultured in tissue culture flasks. The cells were subcultured every 3 to 4 days under continuous G418 selection. During the culturing process, clones with keratinocyte-like morphology and HPV58E7 expression were maintained for more than 30 passages (>P30). These cell lines were used for cell proliferation, migration and invasion, tumor formation and molecular mechanism analyses. (B) Morphology of the BRK-HPV58E7 prototype (58E7P) and its variants BRK-HPV58E7 T20I/G63S (58E7V1), BRK-HPV58E7 G41R/G63D (58E7V2), and BRK-HPV58E7 T74A/D76E (58E7V3) observed under ×100 magnification. Note that the cells display keratinocyte-like morphology and polygonal shape and grow in monolayers. A cell line transfected with pcDNA3.1 empty vector plus EJ-Ras expressed exogenously (BRK-pcDNA3.1) was included as a negative control. (C) Total cell lysates were collected and subjected to Western blotting with pan-keratin (cytokeratin marker), S100A4 (fibroblast marker), and HPV58E7-specific antibodies.

Stable BRK cell lines constitutively expressing E7 oncoproteins were established, as depicted in Fig. 1A. These primary cells were immortalized due to the presence of HPV58E7 and EJ-Ras oncoproteins and were subcultured continuously to achieve high passage numbers. We also cotransfected vector pcDNA3.1 and EJ-Ras in the cells as a negative control. These cells were grown as a monolayer in culture vessels for more than 30 passages. During subculturing, we carefully selected clones of cells that resembled keratinocytes. Morphologically, these cells displayed a polygonal shape (Fig. 1B). We examined the expression of epithelial and fibroblast markers of these cells using pan-keratin and fibroblast-specific protein 1 (FSP1) or S100A4; routinely, our results showed that these cells were epithelial cells and were not fibroblasts (Fig. 1C). HPV58E7 oncoproteins were also detected in these cells (Fig. 1C). The generated cell lines BRK-pcDNA3.1, BRK-HPV58E7 prototype (58E7P), BRK-HPV58E7 T20I/G63S (58E7V1), BRK-HPV58E7 G41R/G63D (58E7V2), and BRK-HPV58E7 T74A/D76E (58E7V3) were used in subsequent assays to compare their abilities in proliferation, invasion, and in induction of tumor growth in athymic nude mice.

### HPV58E7 prototype and V1 induced higher cell proliferation.

**FIG 2:**
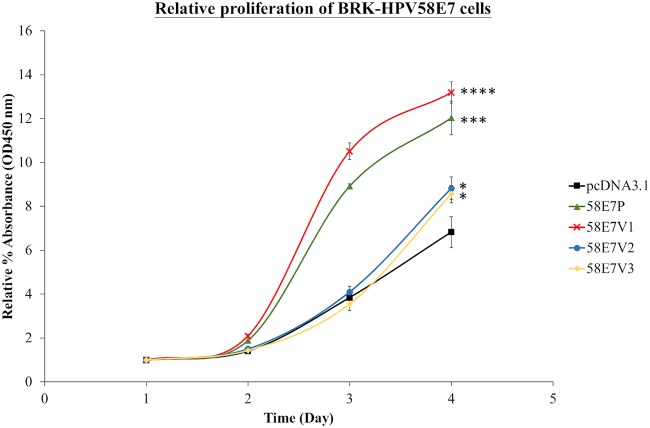
The ability of the HPV58E7 prototype and its variants to induce cell proliferation. Cell proliferation of the BRK-HPV58E7 prototype (58E7P), BRK-HPV58E7 T20I/G63S (58E7V1), BRK-HPV58E7 G41R/G63D (58E7V2), and BRK-HPV58E7 T74A/D76E (58E7V3) was assessed via a Cell Counting Kit-8 (CCK-8) cell viability assay. BRK-pcDNA3.1 (pcDNA3.1) cells were included as a negative control. Cells were seeded into 96-well plates and allowed to grow for 4 days. Relative cell proliferation is the optical density at 450 nm (OD_450_) measured daily as a percentage of the OD reading at day 1. All data are presented as means ± SEM obtained from triplicate culture wells per cell type from three independent experiments. *, *P* < 0.05; ***, *P* < 0.001; ****, *P* < 0.0001.

Having shown that the selected cell lines exhibited stable expression of HPV58E7, we proceeded to investigate their proliferation rates using a Cell Counting Kit-8 (CCK-8) assay. BRK-pcDNA3.1 was incorporated as a negative control. The CCK-8 assay is a colorimetric assay measuring the ability of viable cells to reduce water-soluble tetrazolium salt (WST-8) to an orange-colored formazan product. Our results (Fig. 2) showed that HPV58E7 induced significantly higher cell proliferation than a vector control. Proliferation of 58E7V1 cells was the highest (13.2% ± 0.49%; *P* < 0.0001), followed by that of 58E7P (12.03% ± 0.76%; *P* < 0.001), 58E7V2 (8.83% ± 0.52%; *P* < 0.05), 58E7V3 (8.56% ± 0.39%; *P* < 0.05), and the pcDNA3.1 vector control (6.83% ± 0.70%). Overall, the HPV58E7 prototype and V1 demonstrated abilities which were higher than those of V2 and V3 in inducing proliferation of primary murine cells.

### BRK cells expressing HPV58E7 V1 achieved a greater ability to migrate and invade.

**FIG 3:**
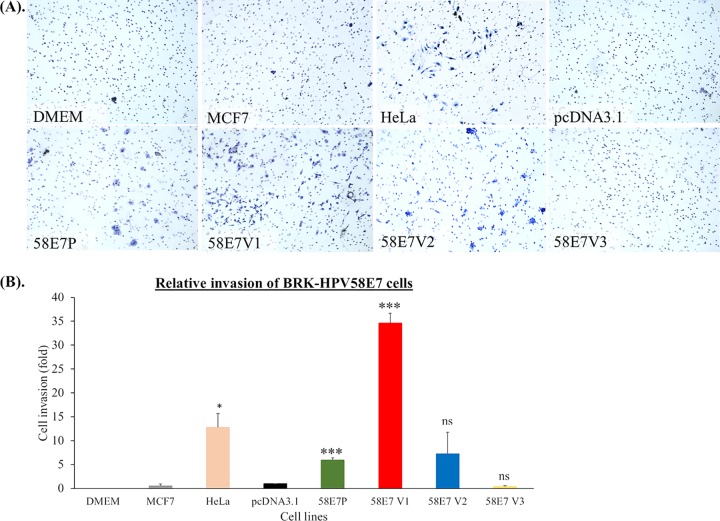
The ability of HPV58E7 prototype and its variants to induce cell migration and invasion. The ability of the BRK-HPV58 E7 prototype (58E7P), BRK-HPV58E7 T20I/G63S (58E7V1), BRK-HPV58E7 G41R/G63D (58E7V2), and BRK-HPV58E7 T74A/D76E (58E7V3) cells to migrate and invade was assessed by a transwell invasion assay. BRK-pcDNA3.1 (pcDNA3.1) and MCF-7 were included as negative controls, while HeLa served as a positive control. (A) Cells that invaded through the Matrigel-coated membrane were stained using hematoxylin. Representative images of the invading cells were taken at a ×400 magnification. (B) Bar graph showing fold change of invading cells relative to that of pcDNA3.1 control cells, calculated from at least three independent fields of view. The experiments were performed in triplicate. Data are presented as means ± SEM. *, *P* < 0.05; ***, *P* < 0.001; ns, not significant.

Having shown that there are differences in the rates of proliferation induced by the HPV58E7 variants, we proceeded to assess the migratory and invasion activities of the HPV58E7 variant-expressing cells using a transwell Matrigel invasion chamber. This assay measures the ability of cells to migrate and invade through a Matrigel matrix, which mimics the extracellular matrix. The migration and invasion activities were highest for 58E7V1 (34.6- ± 2.0-fold; *P* < 0.001), followed by 58E7P (5.9- ± 0.5-fold; *P* < 0.001); and both had activity levels significantly higher than those of the pcDNA3.1 control cells (Fig. 3A and B), while the activities of 58E7V2 (7.2- ± 4.5-fold; *P* > 0.05) and V3 (0.4- ± 0.2-fold; *P* > 0.05) were similar to those of the control. As expected, MCF-7, which was included as a negative control, did not invade. These results show that 58E7V1 has a greater ability to migrate and invade through the extracellular matrix than the 58E7 prototype, V2, and V3.

### HPV58E7 V1 shows greater ability to induce tumor formation in athymic nude mice than the prototype and other variants.

**FIG 4:**
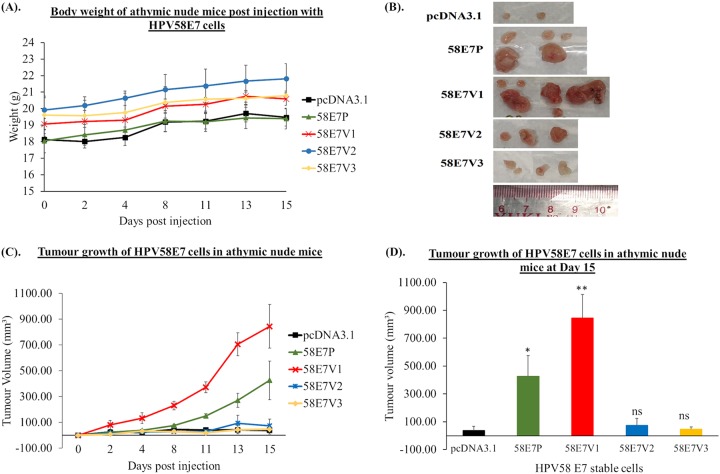
The ability of the HPV58E7 prototype and its variants to induce tumor formation in athymic nude mice. BRK-HPV58E7 prototype (58E7P), BRK-HPV58E7 T20I/G63S (58E7V1), BRK-HPV58E7 G41R/G63D (58E7V2), and BRK-HPV58E7 T74A/D76E (58E7V3) cells were injected into flanks of female athymic nude mice subcutaneously. (A) Relative changes in body weight of athymic nude mice injected with the corresponding cells was monitored throughout the study period. (B) Representative images of tumors excised from athymic nude mice at day 15 postinjection. Note that 58E7P and 58E7V1 cells formed large main tumors, and smaller tumors were found in the vicinity of the main tumors. Also note that 58E7V1 cells formed lobular tumors. (C) Tumor growth of the cells was monitored every other day throughout the study period. Tumor volume was calculated using the formula (*ab*^2^) × 0.5236. (D) Bar graph showing fold change of tumor growth in athymic nude mice relative to that of the pcDNA3.1 at day 15. The experiment was performed in triplicate. Data are presented as means ± SEM. *, *P* < 0.05; **, *P* < 0.01; ns, not significant.

As the 58E7V1 cells showed the greatest proliferation, migration, and invasion ability *in vitro* among the variants tested, we wanted to investigate whether these observations could be translated into increased tumor formation in an *in vivo* model. The general health and body weight of the mice were found to be unaffected during the 2 weeks of the study period (Fig. 4A). We observed that 58E7V1 (854 ± 169 mm^3^; *P* < 0.01) and 58E7P (426 ± 149 mm^3^; *P* < 0.05) cells formed at least 10-fold-larger tumors in the athymic nude mice than the pcDNA3.1 vector control (39 ± 29 mm^3^) (Fig. 4B to D). In addition, the 58E7V1 cells formed 2-fold-larger tumors than those induced by 58E7P. V1 tumors were lobular, whereas those of the prototype were more regular and rounder (Fig. 4B). We also found small tumors in the vicinity of the main tumors in mice injected with both 58E7P and 58E7V1. Interestingly, in mice injected with 58E7V1, but not in those injected with 58E7P, we found some of these small tumors attached to muscle tissue. These *in vivo* observations corroborated our *in vitro* observation that 58E7V1 has a greater ability to migrate and invade. On the other hand, we observed that 58E7V2 (74 ± 50 mm^3^; *P* > 0.05) and V3 (47 ± 16 mm^3^; *P* > 0.05) cells formed smaller tumors in athymic nude mice which were comparable to those of control cells (Fig. 4B to D). Taken together, these results indicate that 58E7V1 has a greater ability to promote tumor formation in athymic nude mice than the 58E7 prototype, V2, and V3.

### HPV58E7 V1 activates AKT and K-Ras/ERK signaling pathways.

**FIG 5:**
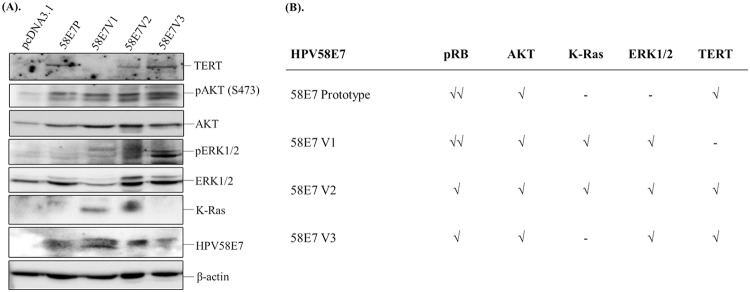
The expression levels of TERT, AKT, K-Ras, and ERK in BRK-HPV58E7 cells. (A) The expression levels of telomerase reverse transcriptase (TERT), phospho-AKT [pAKT (S473)], AKT, phospho-ERK1/2 (pERK1/2), ERK1/2, K-Ras, and HPV58E7 in BRK-HPV58 E7 prototype (58E7P), BRK-HPV58E7 T20I/G63S (58E7V1), BRK-HPV58E7 G41R/G63D (58E7V2), and BRK-HPV58E7 T74A/D76E (58E7V3) cells were examined via Western blotting. (B) The abilities of BRK-HPV58 E7 prototype (58E7P), BRK-HPV58E7 T20I/G63S (58E7V1), BRK-HPV58E7 G41R/G63D (58E7V2), and BRK-HPV58E7 T74A/D76E (58E7V3) cells to degrade pRB (22) and enhance the TERT, AKT, K-Ras/ERK signaling pathways are summarized. √, strength of the perturbation.

Previous studies showed that E7 activates telomerase reverse transcriptase (TERT) (27), AKT (28, 29), and extracellular signal-regulated kinase (ERK) (30) signaling pathways. In view of this, we then wanted to scrutinize the mechanism involved that allows 58E7V1 to achieve greater aggressiveness than the prototype and other variants. Our results (Fig. 5A) showed that, in agreement with previous findings, all HPV58E7 induced increased levels of phosphor-AKT (pAKT) and total AKT. However, we observed that HPV58E7 exhibited different abilities in perturbing different signaling pathways. 58E7V1 induced increased levels of K-Ras and phospho-ERK1/2 but not TERT, while 58E7P, 58E7V2, and 58E7V3 induced elevation of the TERT level. Both 58E7V2 and 58E7V3 increased the level of pERK1/2. Similar to results with 58E7V1, the level of K-Ras was also increased in 58E7V2 cells. The abilities of HPV58E7 to enhance TERT expression and the AKT and K-Ras/ERK signaling pathways, as well as the ability to degrade pRB, as we reported previously (22), are summarized in Fig. 5B. These results indicate that 58E7V1 attains a greater oncogenic potential through activation of AKT and K-Ras/ERK signaling pathways, while the 58E7 prototype, V2 and V3 require increased TERT to maintain proliferation.

## DISCUSSION

The vast majority of studies on HPV focus on HPV16 and HPV18, the two most prevalent cancer-causing types of HPV worldwide. A number of HPV16 and HPV18 intratypic variants have been identified which carry less than 2% variation in genetic sequence from the prototypes (35–37). Of the HPV16 oncogenes, polymorphism is more common in E6 than in E7 (35), and these E6 variants possess differential capacities for driving carcinogenesis (38–40). Similarly, HPV58, which plays an important role in contributing to cervical cancer in East Asia and South America, also carries genetic variations (11, 23, 32) although polymorphisms are more common in E7 than in E6 (23, 32). The genetic variations within HPV58E7 can have a distinct geographical distribution; for instance, HPV58E7 variants found in Columbia (41) are distinct from those found in East Asia (31, 33). HPV58E6, however, appears to be more conserved, and sequence variations lack any differential association with cancer risk (33).

Our epidemiological findings pinpoint a correlation between the HPV58E7 V1 variant and cervical cancer risk (31); however, the evidence is insufficient. An in-depth understanding of how these HPV58E7 variants evoke their differential levels of oncogenicity and drive cancer progression is still lacking. At the molecular level, previous studies have shown that the HPV58E7 prototype can degrade pRB, abrogate the G_1_/S checkpoint, and activate the p53 and DNA damage response pathways (22, 23). However, comparisons of how the HPV58E7 variants perturb these molecular and cellular events have been neglected. In view of this knowledge gap, we decided to compare the oncogenicities of the HPV58E7 variants based on their *in vitro* and *in vivo* activities.

In this report, by using physiologically relevant models, we have further dissected the ability of HPV58E7 V1 to promote the hallmarks of cancers, including cell proliferation, migration, invasion, and tumor formation. We consistently found that 58E7V1 had greater oncogenicity than the prototype and other variants. In our *in vitro* cell proliferation assay, 58E7V1 induced proliferation at a level comparable to that of the 58E7 prototype, as measured by the ability of cells to produce dehydrogenase to reduce WST-8 to formazan dye in culture vessels. This assay allowed us to differentiate the more active 58E7 prototype and V1 variant from the less active V2 and V3. Our subsequent invasion and tumor formation assays then allowed us to compare the oncogenicities of the HPV58E7 prototype and V1 in a more biological setting. Indeed, we found that 58E7V1 could invade through the extracellular matrix and form more aggressive tumors than either the prototype, V2, or V3. These observations are consistent with clinical outcomes as reported in our epidemiological studies (11, 33).

Here, we described for the first time the mechanism of how the HPV58E7 prototype and its natural variants exert their oncogenicities differently. Our previous (22) and current studies highlight that HPV58E7 V1 possesses three key oncogenic features: enhanced ability to degrade pRB, activation of the AKT pathway, and activation of the K-Ras/ERK pathway. These are the key features that allow HPV58E7 V1 to achieve a greater ability to promote the hallmarks of cancers: cell proliferation, immortalization, transformation, migration, invasion, and tumor formation. Even though the HPV58E7 prototype, V2, and V3 possess the ability to promote carcinogenesis, we found that they lack one of the three key features, which compromises their oncogenicities differently. The HPV58E7 prototype degrades pRB to an extent similar to that of HPV58E7 V1, thereby allowing it to promote greater cell proliferation, migration, and invasion than HPV58E7 V2 and V3. However, the HPV58E7 prototype lacks the ability to enhance the K-Ras/ERK signaling axis, which is one of the major oncogenic pathways in HPV-mediated carcinogenesis. This could potentially explain its compromised power to promote the hallmarks of cancer to a degree similar to that of HPV58E7 V1. Despite possessing the ability to enhance TERT, AKT and ERK signaling, these oncogenic features are merely sufficient for HPV58E7 V2 and V3 to achieve high cell proliferation.

For technical consistency, we selected clones of cells that resembled epithelial cells microscopically and expressed a pan-keratin marker and HPV58E7 constitutively. The benefit of using these stable cell lines of murine origin is that this method overcomes the difficulty of achieving stable expression of HPV58E7 in primary keratinocytes. These murine cell lines also produce palpable tumor growth in nude mice within a short duration to allow reproducible comparison among E7 variants. With this approach, we have demonstrated the high oncogenicity of HPV58E7 variants isolated from patients in East Asia. However, polymorphisms in the long control region (LCR) and E6 gene should not be neglected. Future studies should examine the effects of cooperation between the HPV58 LCR, E6, and E7 natural variants to gain a full understanding of how these genetic variations can affect the HPV58 life cycle and the ability of the virus to promote a cancer phenotype using human keratinocytes.

In conclusion, our studies provide a comprehensive understanding of the oncogenic properties of HPV58E7 T20I/G63S, a common natural variant circulating in East Asia, with an increased association with cervical cancer risk. Our laboratory findings reported here support our previous epidemiological and molecular observations. In addition to the greater ability in degrading pRB, we demonstrated for the first time that the HPV58E7 T20I/G63S attains a greater ability to promote cancer progression to eventual carcinoma than the prototype and other natural variants through AKT and K-Ras/ERK activation. The difference in cancer risk conferred by HPV58 variants should be considered in formulating HPV-based cervical cancer screening strategies and in selecting vaccines for East Asian.

## MATERIALS AND METHODS

### Cell lines.

For the invasion assay, HeLa (HPV18-positive cervical cancer) and MCF-7 (HPV-null breast cancer) cells were included as positive and negative controls, respectively. HeLa, MCF-7, HaCaT (HPV-null human keratinocytes), and NIH 3T3 (HPV-null murine fibroblast) cells were purchased from the American Type Culture Collection (ATCC) and maintained in Dulbecco’s modified Eagle medium (DMEM) supplemented with 10% fetal bovine serum (FBS; Gibco) and grown at 37°C in a humidified incubator containing 5% CO_2_. The identities of these human cell lines were validated by short tandem repeat (STR) profiling, using an AmpFlSTR Identifiler Plus PCR amplification kit (ThermoFisher Scientific) with an Applied Biosystems 3500 Series Genetic Analyzer, and analyzed by GeneMapper software, version 5 (Applied Biosystems). The STR profile of all cell lines showed >88% concordance with their reference profiles in the ATCC cell line database.

### Establishment of HPV58E7 stable cell lines.

HPV58E7 stable cell lines were established using primary murine epithelial cells extracted from baby rat kidney (BRK) tissue as described by Massimi and Banks (42) with some modifications. Briefly, BRK cells were extracted from 9-day-old Wistar Hannover rats supplied by the Laboratory Animal Service Centre (LASEC) of the Chinese University of Hong Kong (CUHK). The rats were euthanized by CO_2_ suffocation prior to kidney extraction. HPV58E7 constructs generated in our previous report (22) were transfected into these cells by calcium phosphate precipitation, together with a construct expressing activated H-Ras (EJ-Ras). The cells were maintained in DMEM supplemented with 10% FBS and kept in a 37°C humidified incubator containing 5% CO_2_. The cells were also cultured under a continuous selection of 220 μg/ml Geneticin (G418; ThermoFisher Scientific). Expression profiles of the stable cell lines were monitored at different passages, in which cell pellets were collected and analyzed by Western blotting using an HPV58E7-specific antibody, a pan-keratin antibody, and an S100A4-specific antibody, as described below.

### Cell proliferation assay.

Cell proliferation was determined by water-soluble tetrazolium salt or WST-8 [2-(2-methoxy-4-nitrophenyl)-3-(4-nitrophenyl)-5-(2,4-disulfophenyl)-2H-tetrazolium] assays using a Cell Counting Kit-8 (CCK-8; Dojindo Molecular Technologies, Inc.). HeLa and BRK-HPV58E7 stable cells were seeded into 96-well plates at a density of 2 × 10^3^ cells/well in 100 μl of complete medium (DMEM supplemented with 10% FBS). Cells were allowed to attach and were incubated at 37°C in 5% CO_2_. After 72 h, proliferation was measured by the addition of 10 μl of WST-8 to each well and incubation for a further 2 h at 37°C. Optical density (OD) was measured at 450 nm (OD_450_) using a Victor 3 multilabel plate reader (PerkinElmer).

### Invasion assay.

The migration and invasion abilities of MCF-7, HeLa, and BRK-HPV58E7 stable cells were assayed in 24-well Corning BioCoat Matrigel invasion chambers (Corning), according to the manufacturer’s protocol. Briefly, 5 × 10^4^ cells were resuspended in serum-free DMEM and seeded into the inserts. DMEM supplemented with 20% FBS was added to the lower chambers of the plate as a chemoattractant. After 24 h of incubation at 37°C, the noninvasive cells were removed with cotton swabs. Cells that had migrated through the membrane to the lower surface of the insert were fixed with methanol and stained with hematoxylin. Invading cells were counted from four random fields for each chamber under an upright light microscope at ×400 magnification (Leica). Cell invasion was presented as the fold difference between the number of invading cells over the number of invading BRK-pcDNA3.1 control cells.

### Western blotting.

Total cellular protein was analyzed by lysing cell pellets with 2× sodium dodecyl sulfate (SDS) sample buffer and incubating them at 95°C for 5 min; pellets were then separated by SDS-polyacrylamide gel electrophoresis (SDS-PAGE) and transferred by Western blotting onto a polyvinylidene fluoride membrane (PVDF) (Bio-Rad). The membrane was then probed with the following specific primary antibodies overnight at 4°C: custom-made anti-HPV58 E7 (22); pan-keratin, phospho-AKT (S473), AKT, and K-Ras (Cell Signaling Technology); S100A4 (Abcam); phospho-ERK1/3, ERK1/2, and β-actin (C4; Santa Cruz Biotechnology). Subsequently, the immunoblots were incubated with the appropriate anti-mouse or anti-rabbit horseradish peroxidase (HRP)-conjugated secondary antibodies. Blots were visualized using Clarity Western ECL substrate (Bio-Rad), and images were captured using a ChemiDoc imaging system (Bio-Rad). β-Actin served as a loading control for the blots.

### *In vivo* mouse model.

The experimental procedure was conducted in compliance with animal license regulations and ethics according to the Department of Health, Hong Kong, and Animal Experimental Ethics Committee, CUHK, respectively. Female athymic nude mice, 7 to 8 weeks old (3 mice per group), were purchased from LASEC, CUHK. Tumor models were established in athymic nude mice by injecting approximately 3 × 10^6^ cells (resuspended in 200 μl of DMEM) of each of the BRK-HPV58E7 stable cell lines (BRK-HPV58E7 prototype [58E7P], BRK-HPV58E7 V1 [58E7V1], BRK-HPV58E7 V2 [58E7V2], BRK-HPV58E7 V3 [58E7V3], and BRK-pcDNA3.1 [pcDNA3.1]) into the flank. The animals’ body weight and tumor size were monitored and recorded every other day for 2 weeks. Tumor volume was estimated using the formula (*ab*^2^) × 0.5236, where *a* refers to the largest dimension of the tumor and *b* is the perpendicular diameter (43).

### Statistical analysis.

Results presented are from three independent experiments performed in triplicate. Statistical analyses were performed using GraphPad Prism, version 7, and data are reported as means ± standard errors of the means (SEM). The proliferation, invasion, and tumor formation of the HPV58E7 variant-expressing cell lines were compared with those of the HPV58E7 prototype-expressing cells using *t* tests. A *P* value of <0.05 was considered statistically significant.
